# Association between surgical extent and recurrence in unilateral intermediate- to high-risk papillary thyroid cancer

**DOI:** 10.1186/s12885-023-11307-1

**Published:** 2023-09-18

**Authors:** Siyuan Xu, Hui Huang, Huilei Dong, Xiaolei Wang, Zhengang Xu, Shaoyan Liu, Jie Liu

**Affiliations:** 1 Department of Otolaryngology Head and Neck Surgery, Key Laboratory of Otolaryngology Head and Neck Surgery (Capital Medical University), Ministry of Education, Beijing Tongren Hospital, Capital Medical University, Beijing, 100730 China; 2 Department of Head and Neck Surgical Oncology, National Cancer Center, National Clinical Research Center for Cancer/Cancer Hospital, Chinese Academy of Medical Sciences and Peking Union Medical College, Beijing, 100021 P.R. China; 3 Department of Head and Neck surgery, Cancer Hospital of China Medical University, Liaoning Cancer Hospital & Institute, Shenyang, 110042 Liaonng Province China

**Keywords:** Papillary thyroid carcinoma, Lobectomy; total thyroidectomy, Recurrence-free survival

## Abstract

**Background:**

Guidelines recommend total thyroidectomy (TT) to facilitate radioactive ablation and serological follow-up for intermediate- to high-risk papillary thyroid carcinoma (PTC). However, the association between surgical extent and tumor recurrence in these patients has not been well validated. We aimed to examine the association between the extent of surgery and recurrence in patients with completely resected unilateral intermediate- to high-risk PTC.

**Methods:**

Patients with completely resected unilateral PTC from 2000 to 2017 in a single institute were reviewed. Those who had extrathyroidal extension (ETE) or lymph node metastasis (LNM, cN1 or pN1 > 5 lymph nodes involved) were included for analysis. Cox proportional hazards models were applied to measure the association between surgical extent and recurrence-free survival (RFS) while adjusting for patient demographic, clinicopathological and treatment variables.

**Results:**

A total of 4550 patients (mean[SD] age, 43.0[11.7] years; 3379 women[74.3%]) were included. Of these patients, 2262(49.7%), 656(14.4%), 1032(22.7%), and 600 (13.2%) underwent lobectomy, TT, lobectomy + neck dissection (ND) and TT + ND, respectively. With a median follow-up period of 68 months, after multivariate adjustment, lobectomy was associated with a compromised RFS compared with other surgical extents (HR[95%CI], TT 0.537[0.333–0.866], P = 0.011, lobectomy + ND 0.531[0.392–0.720] P < 0.0001, TT + ND 0.446[0.286–0.697] P < 0.0001). RFS was similar between the two extents with ND (lobectomy + ND, HR [95%CI], 1.196 [0.759–1.885], P = 0.440).

**Conclusion:**

Lobectomy alone is associated with an elevated recurrence risk in patients with unilateral intermediate- to high-risk PTC compared with larger surgical extents. However, lobectomy and ND may provide similar tumor control compared with the conventional approach of TT and ND.

## Introduction

Papillary thyroid carcinoma (PTC) represents the most common histological subtype of all thyroid malignancies and is commonly associated with a good prognosis [1–3]. The treatment modalities for PTC include surgical resection, subsequent radioactive iodine (RAI) ablation and thyrotropin (TSH) suppression. Among the treatment processes, the extent of surgery – either lobectomy or total thyroidectomy (TT) – has always been a topic of debate. The question may also be considered as controversial between a strategy of removing all visible disease and additional management of subclinical lesions.

According to the current guidelines, decision-making is recommended to consider patients’ risks of recurrence and disease-related mortality. Removing all disease is sufficient for patients with a low risk of recurrence and mortality, and surgery plus RAI is advocated otherwise due to the potential benefit of decreasing recurrence and mortality [4, 5]. However, direct evidence is lacking for this recommendation. First, when complete resection can be achieved, the disease-related mortality rate is very low in a majority of these patients (stage I, 10-year disease-related mortality rate < 1%) [6–8]. Second, even if in patients with intermediate- to high-risk groups, the magnitude of recurrence-free survival (RFS) decrease is not clear, considering the lower complication rate of lobectomy, limited magnitude may be accepted for consideration.

It is indisputable in some circumstances that RAI is essential, as surgery is not sufficient to remove all visible disease, such as bilateral lesions, distant metastasis or unresectable disease (T4b). Moreover, the surgical extent of the neck may also influence the recurrence risk together with the extent of the thyroid. A comprehensive consideration of initial risk stratification and probability of tumor control by surgical resection may be useful in the decision-making of treatment.

Thus, we designed the present study of patients with completely resected intermediate- to high-risk PTC to examine the effect of surgical extent on tumor recurrence in these patients.

## Materials and methods

### Patients

This retrospective cohort study included untreated adult patients (18–75 years) who underwent surgery for PTC at the authors’ institute from January 2000 to March 2017. Data analysis was performed from May 1 to 20, 2022. The study was approved by the Ethics Committee of the Cancer Hospital, Chinese Academy of Medical Sciences. Informed consent was obtained at the time of surgery for the general use of clinical information for future studies. The study followed the Strengthening the Reporting of Observation Studies in Epidemiology (STROBE) reporting guidelines for observation studies.

Patients with extrathyroidal extension (ETE) or lymph node metastasis (LNM, cN1 or pN1 > 5 lymph nodes involved) were admitted. The initial risk stratification were performed according to the 2015 American Thyroid Association (ATA) guidelines. Patients with bilateral primary tumors, evidence of contralateral lymph node or distant metastasis, incomplete tumor resection, aggressive histologic variants or insufficient follow-up data (less than 24 months) were excluded.

Variables such as patient age, sex, tumor characteristics, and treatment modalities were obtained from the medical records. Primary tumor size, ETE, LNM and extranodal extension (ENE) were defined by postoperative pathologic examination. Gross ETE was identified on surgical findings. Standard pathologic diagnoses were based on World Health Organization criteria. The extent of surgery was defined as follows: (1) lobectomy (with or without isthmectomy and ipsilateral central compartment dissection), (2) total thyroidectomy (TT, with or without central compartment dissection), (3) lobectomy + neck dissection (ND, no less than level II-IV) and (4) TT + ND. The primary outcome was RFS and the secondary outcome was disease-specific survival (DSS). Local and regional recurrences were defined as structural disease as determined by either a cytologist or a pathologist. Distant metastasis was defined using computed tomography or emission computed tomography. Thyroid function tests were routinely measured during follow-up, and biochemical abnormalities alone were not recorded as recurrence. During the study period, TT was mainly performed only on patients with bilateral thyroid cancer, contralateral neck metastasis, distant metastasis or unilateral disease with advanced tumor. Therapeutic central compartment dissection was routinely performed and prophylactic central compartment dissection was performed when the surgeon considers a high metastasis risk.

### Statistics

The sample size of the study was reflective of patients meeting eligibility criteria based on their histologic diagnosis and year of treatment. All statistical analyses were performed using IBM SPSS software version 26.0 (IBM Corp., Armonk, NY). Clinicopathologic characteristics were compared across groups using the t test for continuous variables and the Pearson χ2 test for categorical variables. Cox proportional models were applied to examine the association between the extent of surgery and DFS and DSS. Significance levels were interpreted as 2-sided P < 0.05.

## Results

A total of 4550 patients (mean [SD] age, 43.0 [11.7] years; 3379 women [74.3%]) were included in the analysis. Of these patients, 2262 (49.7%), 656 (14.4%), 1032 (22.7%), and 600 (13.2%) underwent lobectomy, TT, lobectomy + ND and TT + ND, respectively. Compared with patients who underwent lobectomy, patients who underwent TT were likely to be female, older, and encompassed more tumors with ETE, multifocality, and ENE (all P < 0.05) (Table 1).

**Table 1:** Patient demographic, clinicopathologic and treatment factors by extent of surgery (2000–2017)

	Lobectomy N = 2262	TT N = 656	P value	Lobectomy + ND N = 1032	TT + ND N = 600	P value
**Female**	1708(75.5%)	528 (80.5%)	0.008	704 (68.2%)	439 (73.2%)	0.035
**Age ≥ 45 yrs**	935(41.3%)	384(58.5%)	< 0.0001	311(30.1%)	236(39.3%)	< 0.0001
**Primary size ≥ 2 cm**	213(9.4%)	66(10.1%)	0.621	266(25.8%)	166(27.7%)	0.404
**ETE**	2079(91.9%)	626(95.9%)	0.001	578(56.0%)	435(72.5%)	< 0.0001
**Gross ETE**	315(13.9%)	111(16.9%)	0.056	166(16.1%)	126(21.0%)	0.013
**Multifocality**	379(16.8%)	206(31.4%)	< 0.0001	215(20.8%)	169(28.20%)	< 0.001
**LNM**	1124(49.7%)	329(50.2)	0.835	1023(99.1%)	591(98.5%)	0.242
**ENE**	135(6.0%)	63(9.6%)	0.001	250(24.2%)	235(39.2%)	< 0.0001
**Central neck dissection**	1963(86.8%)	621(94.7%)	< 0.0001	1032(100%)	600(100%)	1.000
**RAI**	5(0.2%)	60(9.1%)	< 0.0001	3(0.3%)	169(28.2%)	< 0.0001

**Fig. 1:**
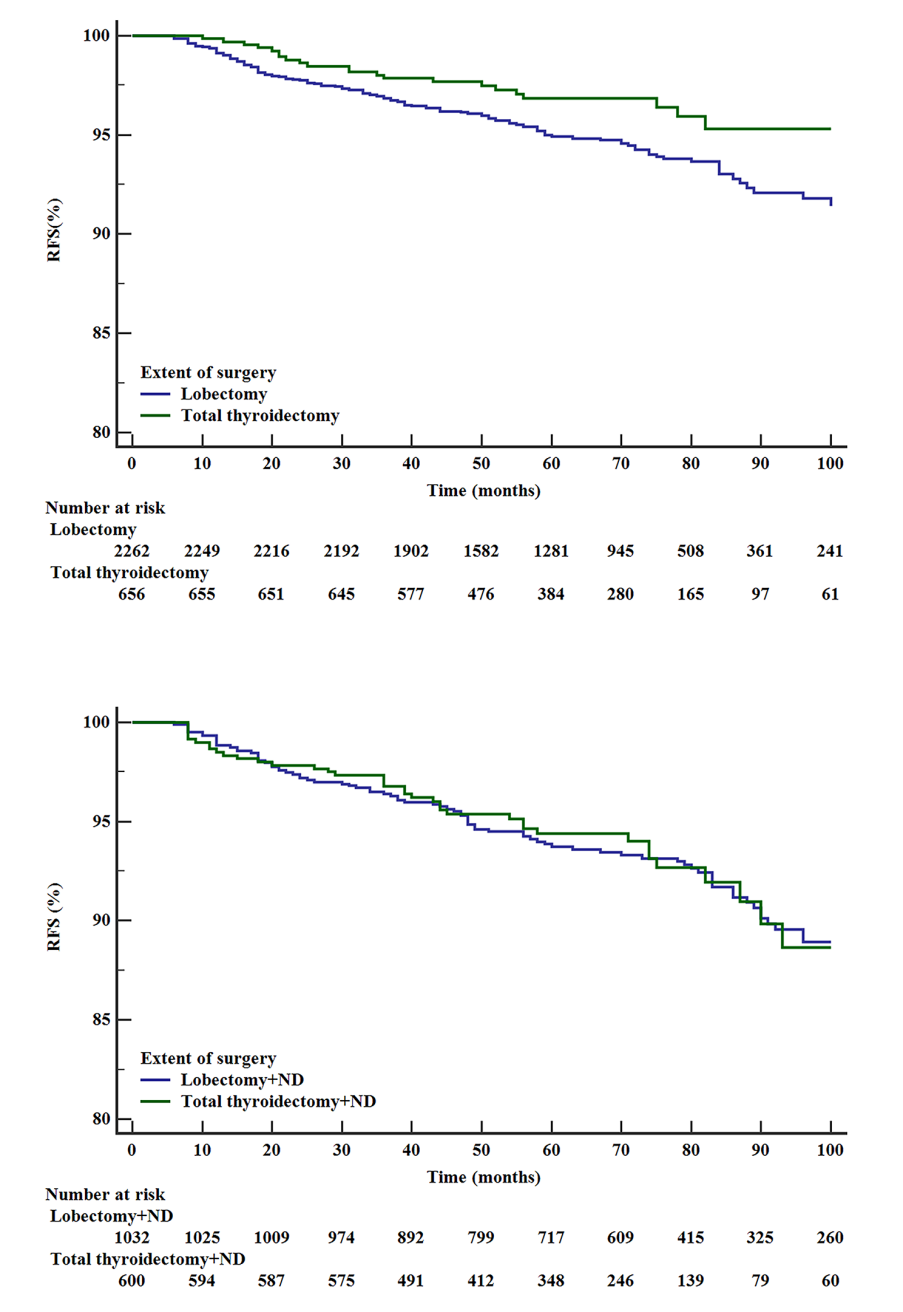
Unadjusted RFS of patients who underwent lobectomy or TT **(1a)**, P = 0.017. Unadjusted RFS of patients who underwent lobectomy + ND or TT + ND **(1b)**, P = 0.750

**Fig. 2:**
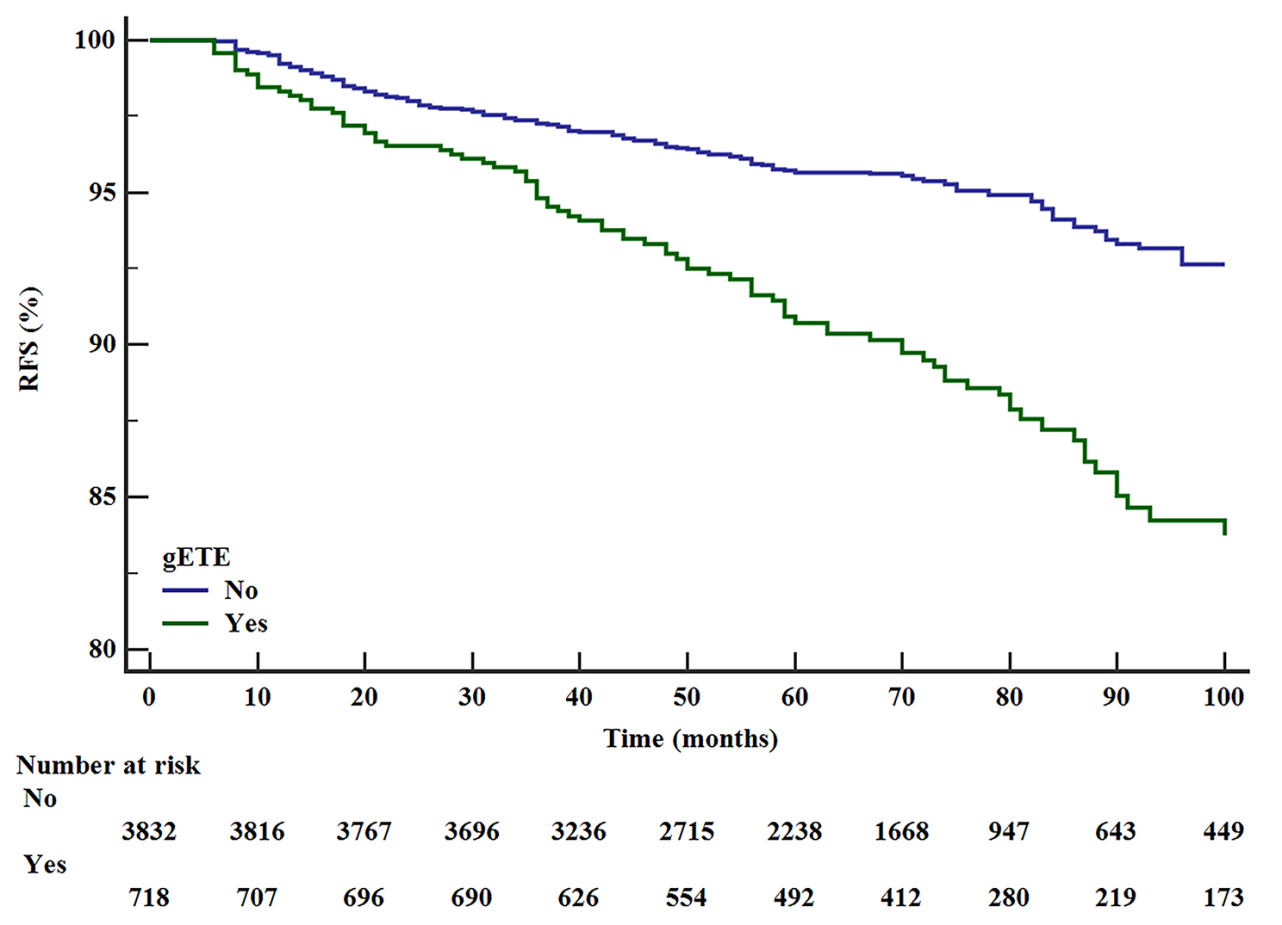
Unadjusted RFS of patients with or without gETE, P < 0.0001

With a median follow-up time of 68 months, unadjusted RFS was slightly better for patients who had TT than for those who had lobectomy at 5 years (96.8% vs. 94.9%) and 8 years. (95.3% vs. 91.8%), P = 0.017 (Fig. 1a), while RFS was similar between patients who underwent TT + ND and lobectomy + ND at 5 years (94.4% vs. 93.7%) and 8 years, (88.6% vs. 88.9%), P = 0.750 (Fig. 1b). g ETE (indication of high-risk PTC) was inversely related to RFS both at 5 years (90.3% vs. 95.3%) and 8 years (82.1% vs. 91.3%), P < 0.0001(Fig. 2).

**Fig. 3:**
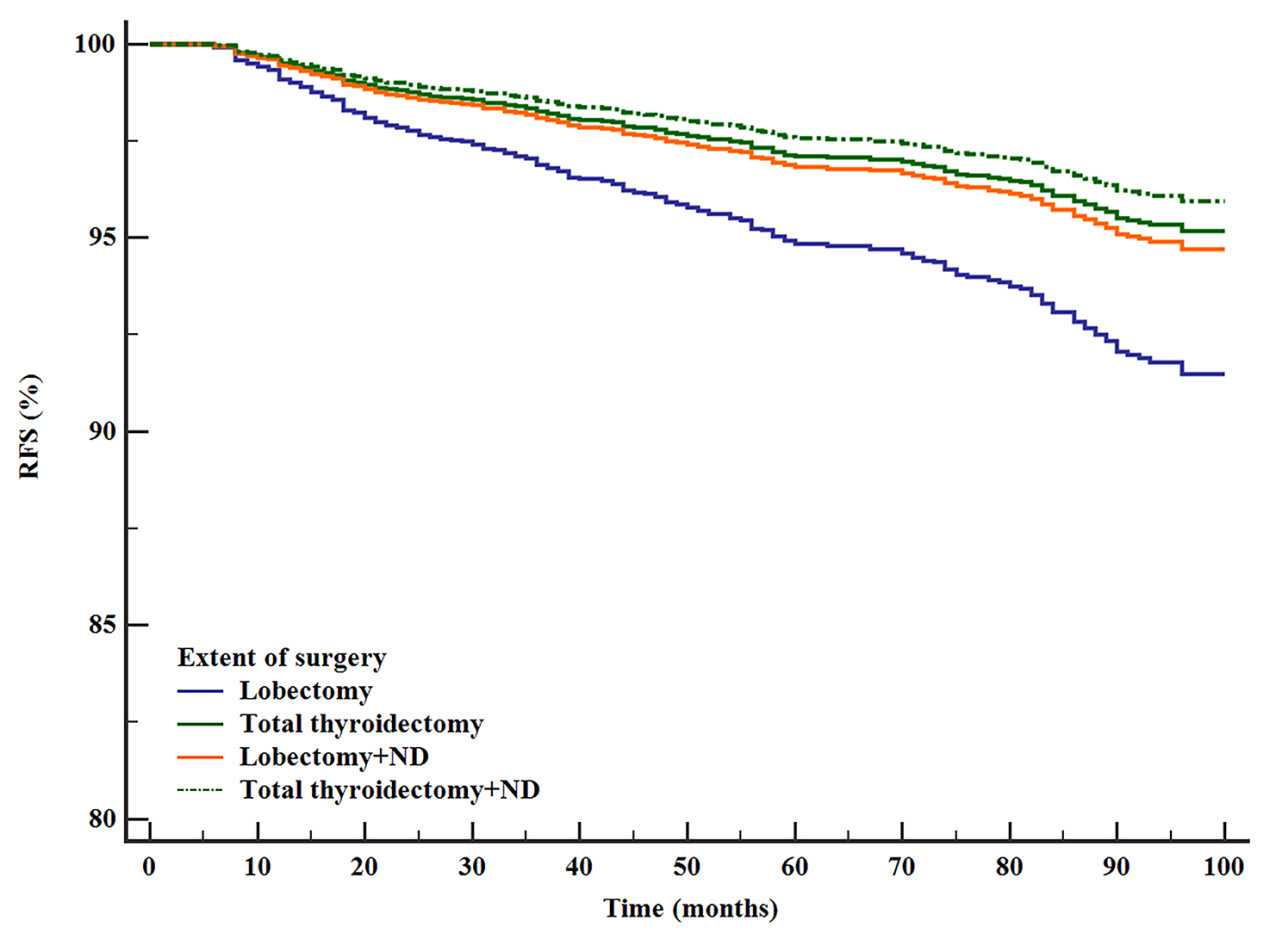
Adjusted RFS of patients with different surgical extents (adjusting sex, patient age, primary size, ETE, g ETE, multifocality, LNM, ENE, central neck dissection and RAI), P < 0.0001

**Fig. 4:**
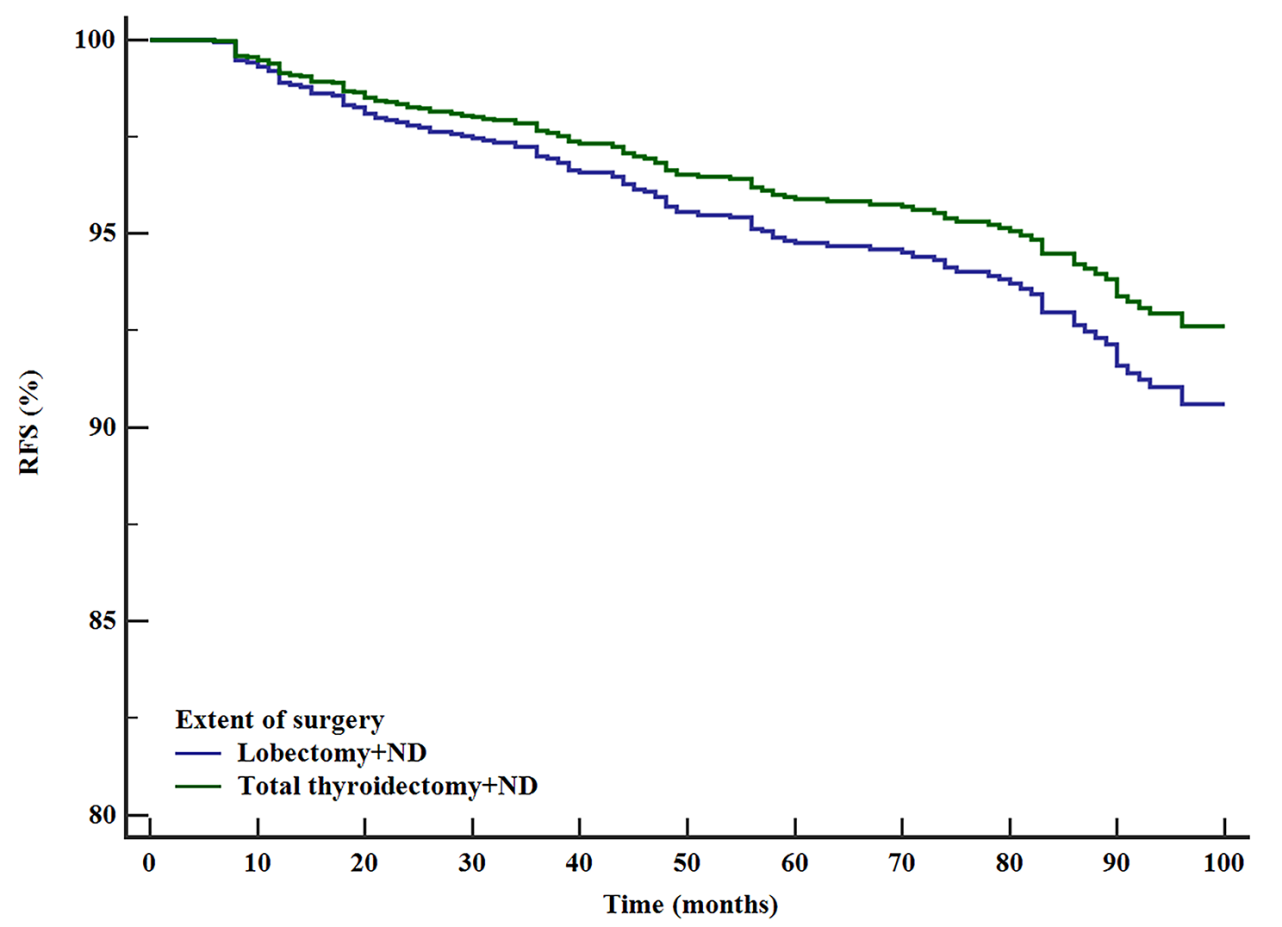
Adjusted RFS of patients who underwent lobectomy + ND or TT + ND (adjusted for sex, patient age, primary size, ETE, gETE, multifocality, LNM, ENE and RAI), P = 0.440

After adjustment for patient demographics, clinicopathologic factors, and other treatment variables, lobectomy was associated with a compromised RFS compared with other surgical extents (HR [95% CI] P value, TT 0.537[0.333–0.866], P = 0.011, lobectomy + ND 0.531[0.392–0.720] P < 0.0001, TT + ND 0.446[0.286–0.697] P < 0.0001) (Table 2) (Fig. 3). RFS was similar between the two extents with ND (lobectomy + ND, HR [95% CI], 1.196[0.759–1.885], P = 0.440) (Table 2) (Fig. 4).

**Table 2:** Cox proportional hazards model of recurrence for patients with intermediate- to high-risk PTC (2000–2017)

Characteristics	Hazard ratio (95% CI)		
	Whole cohort	Patients without ND	Patients with ND
	N = 4550	N = 2918	N = 1632
**Female**	0.812(0.633–1.041)	0.961(0.671–1.377)	0.683(0.482–0.968) *
**Patient age per 10 years**	1.059(0.957–1.172)	0.951(0.820–1.102)	1.161(1.009–1.336) *
**Primary size per 1 cm**	1.518(1.345–1.712) *	1.779(1.4998–2.112) *	1.297(1.095–1.537) *
**ETE**	0.713(0.520–0.977) *	0.629(0.394–1.005)	0.791(0.510–1.226)
**gETE**	1.081(0.753–1.552)	0.718(0.406–1.270)	1.435(0.891–2.314)
**Multifocality**	1.526(1.152–2.023) *	1.605(1.088–2.367) *	1.426(0.946–2.151)
**LNM**	2.648(1.823–3.845) *	2.708(1.768–4.148) *	4.413(0.607–32.100)
**ENE**	1.824(1.378–2.413) *	1.694(1.060–2.707) *	1.849(1.294–2.643) *
**Central neck dissection**	0.796(0.584–1.085)	0.592(0.382–0.918) *	NA
**RAI**	1.036(0.598–1.793)	2.199(0.941–5.137)	0.757(0.384–1.492)
**Extent of surgery**			
Lobectomy	1	1.918(1.135–3.241) *	NA
TT	0.537(0.333–0.866) *	1	NA
Lobectomy + ND	0.531(0.392–0.720) *	NA	1.196(0.759–1.885)
TT + ND	0.446(0.286–0.697) *	NA	1

*Indicates statistical significance with P value ≤ 0.05

Unadjusted 5- and 8-year DSS was similar between patients who had different surgical extents (5-year lobectomy 100%, TT 99.8%, lobectomy + ND 99.9%, TT + ND 100%, 8-year lobectomy 100%, TT 99.8%, lobectomy + ND 99.4%, TT + ND 100%, respectively, P = 0.463).

After stratification by recurrence site, unadjusted metastasis-free survival rates (including both regional and distant) were better in patients who underwent lobectomy + ND than in patients who underwent TT + ND at both 5 years (95.3% vs. 91.8% and 8 years (91.3% vs. 82.5%), P = 0.04, while unadjusted metastasis-free survival rates were similar in patients who underwent lobectomy and TT (5 years 95.5% vs. 96.7%, 8 years 92.2% vs. 94.8%, P = 0.160).

Of patients who underwent lobectomy + ND and lobectomy, 37 (3.6%) and 46 (2.0%) recurrences were identified in the residual thyroid lobes, respectively, and the recurrence risks at 5 years and 8 years were estimated by Kaplan-Meier curves as 2.8%, and 1.8%, and 5.5% and 3.4%, respectively.

## Discussion

In the present study, we examined the association between surgical extent and tumor recurrence in a cohort of patients with intermediate- to high-risk unilateral PTC. After adjusting for patient demographics, clinicopathologic factors, and other treatment variables, it is not surprising that a smaller surgical extent was associated with an elevated recurrence risk. However, it should be noted that the combination of lobectomy and ND achieved similar tumor control compared with the conventional extent for extrathyroidal and metastatic PTC (TT + ND). This result overturns the previous impression that TT may benefit in more serious patients. In fact, it is natural that the treatment strategy also has an important effect on the recurrence risk, e.g., appropriate ND may decrease the regional recurrence risk and obscure the benefit of TT and RAI. Our findings call into consideration that TT may not be mandatory in selected intermediate- to high-risk PTC patients when recurrence risk can be well controlled by initial surgery.

The analysis of recurrence site can partly explain the results. Patients who underwent lobectomy had higher regional and distant recurrence rates than those who underwent TT. In addition to recurrences on the contralateral lobe, lobectomy was associated with a 2- to 3-fold of elevated recurrence risk compared with TT, which conforms with the HR of the study. Additionally, ND well controlled the regional recurrence risk, which induces lobectomy + ND to be noninferior to TT + ND in regard to RFS. Moreover, according to the 95% CI of HR (0.759–1.885), lobectomy + ND is associated with a recurrence elevation of at most 1.9 time that of TT + ND. Considering the absolute recurrence rate of 6% in this study, a recurrence elevation of less than 5% in the lobectomy + ND group is anticipated. As lobectomy is associated with a significantly lower complication risk, the surgical approach (lobectomy + ND) may be considerable in appropriate patients.

The optimal surgical extent for PTC has includes been a topic of debate in recent years. The major concerns of lobectomy for intermediate- and high-risk patients include additional recurrences on the contralateral thyroid lobe and possible recurrence or mortality elevation caused by decreased use of RAI. Although lobectomy was not recommended by guidelines for these considerations, inconsistent data have always existed [9–14]. A nationwide study indicated similar overall survival between patients who underwent lobectomy and TT, which included 8% and 25% patients with ETE and LNM, respectively [11]. Another nationwide analysis by Suman P et al. reported that TT achieved better overall survival than lobectomy in patients with cN1, lymphvascular invasion, ETE, positive margins, poorly differentiated PTC or M1 disease after propensity score matching (5 years: 96.5% vs. 94.7% and 10 years: 90.9% vs. 88.5%, respectively, P = 0.031) [12]. In the present study, only patients with PTC that is feasible for complete resection were admitted. The disease-related mortality rate is extremely low (< 1%), so multivariate analysis of DSS is not possible to be applied, but its difference between the lobectomy and TT groups is acceptable.

The limitations of our study include those inherent to single-institute studies. Several specific management factors exist and may influence the final results, e.g., a low rate of RAI ablation (approximately 30% among patients who had TT) which seems not be guideline conformed. The potential benefit of RAI for patients without detectable residual tumor is debatable. According to the multivariate analysis of the study, RAI was not significantly associated with the outcome. The reason may be that the primary outcome is structural recurrence. Except for its potential effect of controlling subclinical lesions, RAI may also increase the result event with its early detection by whole-body scan. Second, due to the retrospective nature of the study, some factors were not available in the database, such as detailed lymph node diameter, vascular invasion, BRAF status and surgical complication. The follow-up period was not long enough to detect most disease-related mortality. Then, the survival result was not designed as the primary outcome of the study.

Despite the limitations, our study provides valuable information regarding the outcomes of thyroid lobectomy for extrathyroidal or metastatic PTC, and we identified that lobectomy and ND may provide similar effects on tumor control in selected intermediate- to high-risk patients. This indicates that surgical extent should be considered in addition to initial risk stratification in decision-making of the treatment choice.

## Conclusion

Lobectomy alone is associated with an elevated recurrence risk in patients with unilateral intermediate- to high-risk PTC compared with other surgical extents. However, lobectomy and neck dissection may provide similar tumor control compared with conventional total thyroidectomy and neck dissection.

## Data Availability

The datasets used and/or analysed during the current study available from the corresponding author on reasonable request.
